# Tracking Photodynamic- and Chemotherapy-Induced Redox-State Perturbations in 3D Culture Models of Pancreatic Cancer: A Tool for Identifying Therapy-Induced Metabolic Changes

**DOI:** 10.3390/jcm8091399

**Published:** 2019-09-06

**Authors:** Mans Broekgaarden, Anne-Laure Bulin, Jane Frederick, Zhiming Mai, Tayyaba Hasan

**Affiliations:** Wellman Center for Photomedicine, Department of Dermatology, Harvard Medical School and Massachusetts General Hospital, 40 Blossom Street, Boston, MA 02114, USA (M.B.) (A.-L.B.) (J.F.) (Z.M.)

**Keywords:** oncology, oxidative phosphorylation, metabolomics, reactive oxygen species, automated image analysis, two-photon excited fluorescence microscopy

## Abstract

The metabolic plasticity of cancer cells is considered a highly advantageous phenotype that is crucial for disease progression and acquisition of treatment resistance. A better understanding of cancer metabolism and its adaptability after treatments is vital to develop more effective therapies. To screen novel therapies and combination regimens, three-dimensional (3D) culture models of cancers are attractive platforms as they recapitulate key features of cancer. By applying non-perturbative intensity-based redox imaging combined with high-throughput image analysis, we demonstrated metabolic heterogeneity in various 3D culture models of pancreatic cancer. Photodynamic therapy and oxaliplatin chemotherapy, two cancer treatments with relevance to pancreatic cancer, induced perturbations in redox state in 3D microtumor cultures of pancreatic cancer. In an orthotopic mouse model of pancreatic cancer, a similar disruption in redox homeostasis was observed on ex vivo slices following photodynamic therapy in vivo. Taken together, redox imaging on cancer tissues combined with high-throughput analysis can elucidate dynamic spatiotemporal changes in metabolism following treatment, which will benefit the design of new metabolism-targeted therapeutic approaches.

## 1. Introduction

Pancreatic ductal adenocarcinoma (PDAC) continues to have a particularly poor prognosis due to limited effective treatment options [1]. Moreover, PDAC is typified by high degrees of metabolic diversity and plasticity [2], which allows cancer cells to adjust their metabolism to fluctuations in local and temporal nutrient availabilities [3]. This feature is considered to be highly advantageous in the acquisition of treatment resistance as cells can acquire the necessary energy to engage survival mechanisms [4,5]. Consequently, the clinical exploitation of metabolism-modulating agents is currently being explored as stand-alone or (neo) adjuvant cancer treatments [2]. Within this context, a better understanding of the adaptive metabolic phenotypes of cancer tissues is of critical importance to identify treatments or combination therapies that are best suited to circumvent resistance and achieve favorable outcomes [3,6]. 

Three-dimensional (3D) culture models of cancer embody a promising platform to screen new treatment strategies, as they recapitulate cancer architectures and heterogeneity more faithfully than conventional monolayer cultures [7], additionally resulting in more representative metabolic characteristics [8]. However, biochemical methods for tracking metabolic changes in such models are practically challenging due to lengthy tissue processing, which prohibits the effective recovery of metabolites [9]. A promising procedure to probe the metabolic state in tissue cultures leverages the autofluorescence of reduced nicotinamide dinucleotide (NAD(P)H) and oxidized flavoprotein adenine dinucleotide (FAD) [10,11,12]. Endogenous NAD(P)H and FAD are predominantly found in the mitochondria where they are cycled between oxidized and reduced states through active tricarboxylic acid (TCA) cycle reactions and oxidative phosphorylation (OxPhos) [13]. During active OxPhos or upon imbalanced redox homeostasis, NAD(P)H and FADH_2_ are oxidized to NAD^+^ and FAD, respectively. Through detection of the fluorescence intensities of NAD(P)H (*I^NAD(P)H^*) and FAD (*I^FAD^*), the optical redox ratio (ORR) can be derived by dividing *I^FAD^* by the sum of *I^NAD(P)H^* and *I^FAD^* [13,14]. This normalized metric describes the redox state of the cells or tissues, which is closely linked to their metabolic properties and the abundance of oxidizing or reducing agents. To simplify, the higher the ORR is, the higher the oxidative state of the tissues is. Such redox changes can be caused by the abundance of reactive molecular species, mitochondrial uncoupling, or increased oxidative metabolism.

This imaging method was successfully applied on numerous occasions (e.g., References [10,15,16,17,18,19]) and was extensively validated with biochemical methods [14,20]. However, the application of intensity-based redox imaging to 3D culture models saw limited application, as signal collection is challenged by the 3D architecture of the cultures, the low fluorescence yields of NAD(P)H and FAD, and the high magnifications required to acquire satisfactory signal-to-noise ratios that prevent imaging of whole spheroids/microtumors/organoids. Although redox imaging was performed on whole cancer spheroids with fluorescence lifetime imaging [10], such instrumentation is not widely available in biological laboratories. Thus, achieving intensity-based redox imaging at low magnifications with conventional fluorescence microscopy could be immensely useful to enable more accessible exploratory investigations on cancer metabolism in 3D culture models. 

In this study, we adapted established methods for intensity-based redox imaging and combined it with high-throughput automated image analysis to specifically investigate the heterogeneity and adaptability of redox metabolism in whole microtumors, generating hundreds of readouts per experiment. The novelty in this study is reflected in the combination of (1) the acquisition of NAD(P)H and FAD fluorescence intensities at low magnifications to image numerous microtumors within single images, (2) the application of readily available image analysis tools to generate multiple relevant readouts for individual microtumors in each image, and (3) its combination with a recently developed image-based assay for multiparametric assessment of treatment effects. This integrated workflow presents a highly useful approach to assess the effects of cancer therapies on the metabolism and vice versa. Here, we applied our adapted redox imaging and image analysis methodology to characterize metabolic heterogeneity among 3D culture models established from different PDAC cell lines, and identified treatment-induced alterations in metabolic/redox states that were recapitulated in vivo. The methodologies and research findings presented here encourage and enable further investigations toward adaptive cancer metabolism in both preclinical and clinical cancer tissues, such as patient-derived organoid models, and explore new avenues for therapy development.

## 2. Experimental Section

### 2.1. Chemicals and Reagents

All cell culture reagents were purchased from Corning (Tewksbury, MA, USA), except for fetal bovine serum (FBS; Gibco, Thermo Fisher Scientific, Waltham, MA, USA). Sodium azide (NaN_3_), 2,4-dinitrophenol (DNP), benzoporphyrin derivative (BPD, verteporfin), NADH, and FAD were purchased from Sigma-Aldrich (St. Louis, MO, USA). Oxaliplatin and rotenone were obtained from Cayman Chemicals (Ann Arbor, MI, USA). Hydrogen peroxide (H_2_O_2_, 30%) was purchased from Fisher Chemical (Thermo Fisher Scientific, Waltham, MA, USA). 

### 2.2. Cell Culture

Human pancreatic cancer cell lines MIA PaCa-2 (CRL-1420), PANC-1 (CRL-1469), and AsPC-1 (CRL-1682) were obtained from the American Type Culture Collection (ATCC, Manassas, VA, USA) between 2011 and 2015. Cell lines were authenticated based on morphological inspection and comparison to the vendor’s description. CAF6 cells were provided by Dr. Sandro Goruppi and Dr. Gian Paolo Dotto (Cutaneous Biology Research Center, Massachusetts General Hospital). MIA PaCa-2 and PANC-1 cells were maintained in Dulbecco’s modified Eagle medium (DMEM), and AsPC-1 cells were cultured in Roswell Park Memorial Institute (RPMI) 1640 medium. All culture media contained 10% (*v/v*) FBS, 5 mM glutamine, and 1% (*v*/*v*) penicillin/streptomycin. All cell lines were typically passaged weekly at a 1:8 ratio and maintained in standard culture conditions (37 °C, 5% CO_2_, 95% air). Cells were typically discarded after passage 30. Throughout the experiments, all lines were confirmed mycoplasma-free as assessed using the MycoAlert Plus mycoplasma detection kit (Lonza, Portsmouth, NH, USA). 

### 2.3. Suspended Spheroid Cultures of Pancreatic Cancer Cell Lines

Suspended 3D cultures were established from AsPC-1, MIA PaCa-2, and PANC-1 cell lines. Throughout this study, these suspended 3D cultures are referred to as cancer spheroids. Cells were seeded at a density of 2500 cells/well (50 µL of a 50,000 cells/mL suspension) in U-bottom ultra-low-adhesion 96-well plates (Costar, Corning, Kennebunk, ME, USA). Spheroids were formed during 48-h of incubation in standard culture conditions, i.e., until culture day 3. Spheroid areas at the time of measurement (day 5) were 1.15 × 10^6^ ± 9.20 × 10^4^ µm^2^ for AsPC-1, 9.39 × 10^5^ ± 1.13 × 10^5^ µm^2^ for MIA PaCa-2, and 7.78 × 10^5^ ± 2.86 × 10^5^ µm^2^ for PANC-1 

### 2.4. Evaluation of Redox States under Controlled Redox Conditions in Spheroid Cultures

Rotenone (5 µM), sodium azide (250 µM), DNP (5 mM), and hydrogen peroxide (2 mM) were added on day 3. Two-photon laser scanning microscopy was performed after 48 h of incubation (on day 5), as described in Section 2.5. To evaluate the toxicity of the treatments, similar 48-h incubations were performed with rotenone (2.5, 5, and 10 µM), sodium azide (125, 250, and 500 µM), DNP (2.5, 5, and 10 mM), and H_2_O_2_ (1, 2, and 4 mM). Toxicity was evaluated directly following the 48-h exposure (day 5) by quantification of spheroid viability using the CALYPSO (comprehensive analysis of treatment effects in architecturally complex organotypic models) method [21].

### 2.5. Two-Photon Excited Fluorescence Microscopy

**Figure 1:**
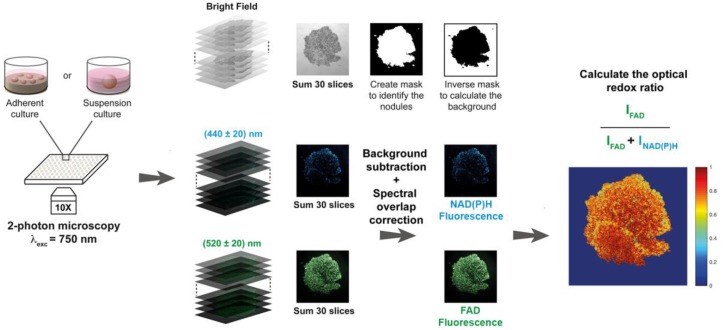
Schematic representation of the workflow for image acquisition and subsequent analysis (NAD(P)H = reduced nicotinamide dinucleotide (phosphate), FAD = flavin adenine dinucleotide). Image acquisition was performed using a multiphoton laser scanning microscope delivering a 750-nm excitation (alternatively, a single-photon excitation (405 nm) may be used [18] on a confocal set-up). A z-stack of 30 images (0.1 µm step-size) was measured to increase the signal-to-noise ratio while preventing photothermal effects. The image analysis procedure firstly summed the images within the z-stack. Bright-field images were used to highlight the individual spheroids/microtumors using a binary mask, of which the inverse was used to determine the median background fluorescence intensities. Background was subtracted from the summed fluorescence images, after which the redox ratio was calculated on a pixel-by-pixel basis. Median redox ratios and redox ratio heatmaps were used as primary outputs.

NAD(P)H and FAD autofluorescence intensities were measured using an Olympus FV1000 multi-photon fluorescence microscope through a 10× objective (air, 0.4 NA) allowing a field of view of 1272.32 × 1272.32 µm^2^. Then, 750-nm pulsed excitation was delivered using a MaiTai DeepSee red/infrared (IR) laser unit tunable between 690 and 1040 nm (Spectra-Physics, Santa Clara, CA, USA). NAD(P)H and FAD autofluorescence signals were collected by two photomultiplier tubes with individual band-pass filters, 440 ± 20 nm and 520 ± 20 nm for NAD(P)H and FAD, respectively, with a scanning speed of 12.5 µs/pixel. By integrating the autofluorescence intensities in both channels over a z-stack of 30 images acquired with 0.1-µm intervals, photothermal effects were minimized, and satisfactory signal-to-noise ratios were achieved (Figure 1). Laser and image acquisition parameters were kept identical throughout all experiments. It should be noted that single-photon excitation can also be performed instead of two-photon excitation, using a 405-nm excitation source. 

### 2.6. Image Analysis

A customized Matlab-based code was developed to calculate the ORR, which can be made available upon request. Firstly, the raw images from the 30-image z-stack acquired with each channel (bright-field and the two fluorescent channels) were summed to substantially increase the signal-to-noise ratio. Secondly, a mask highlighting each individual object present in the field of view was created in the form of a binary image: The bright-field image was binarized using a locally calculated threshold value (adaptive threshold) to overcome inhomogeneous illumination during image acquisition. Through this method, individual spheroids/microtumors were accurately identified and indexed.

The background fluorescence intensities were then calculated for individual channels by applying the inverse bright-field mask to the summed fluorescence images for every channel. It was found that the most reliable method to establish the background intensities consisted of calculating the median pixel intensity within the inverse mask of the fluorescence images (i.e., intra-microtumor space). Background intensities were subsequently subtracted from every pixel within the fluorescence images.

To establish the spectral overlap between the NADH and FAD acquisition bandwidths following 750-nm two-photon excitation, standard curves of NADH and FAD (0.01–1 mM in milliQ-grade water, 100 µL/well) were prepared and imaged as described above. Following z-stack summing, it was found that FAD fluorescence could not be detected at 440 ± 20 nm, yet there was substantial emission of NADH detected at 520 ± 20 nm (Figure S1A,B). At physiologically relevant concentrations (0.05–0.2 mM [22]), the NADH emission detected at 520 ± 20 nm approximated 39% of the emission intensity detected at 440 ± 20 nm. Thus, a correction factor of 0.39 was used to correct for spectral overlap of NADH emission in the FAD detection bandwidth: *Corrected I^FAD^* = *I^FAD^* − (0.39 × *I^NAD(P)H^*).

As the photosensitizer that was used for photodynamic therapy (PDT), BPD has fluorescent properties at various wavelengths [23]. Solutions with a concentration range of BPD were prepared and imaged as described above to ensure that no BPD fluorescence was detected with the NAD(P)H or FAD detection settings. The results confirmed that BPD does not interfere with the detection of NAD(P)H or FAD at the defined detection settings and does not influence the optical redox ratio calculations (Figure S1C). 

Lastly, following masking and background correction, the ORR was calculated on a pixel-by-pixel basis by using the following ratio: ORR = *Corrected I^FAD^*/(*I^NAD(P)H^* + *Corrected I^FAD^*). This ratio is contained within the 0–1 interval and its value can be represented on a heatmap to provide information on the spatial distribution of the ORR throughout individual nodule architectures. A single overall ORR value was calculated using the averaged values of *I^NAD(P)H^* and *Corrected I^FAD^*. 

### 2.7. Establishing Adherent 3D Cultures of Pancreatic Cancer

Adherent 3D cultures were established on solidified growth factor-reduced Matrigel (lot 5173009, Corning, Tewksbury, MA, USA) in black-walled, glass-bottom 24-well plates (Greiner Bio-One). Matrigel (maintained at −20 °C) was thawed at 4 °C overnight and kept on ice throughout all procedures. Matrigel scaffolds were prepared by dispersing 250 µL in the ice-cold 24-well plates, followed by 20-min incubation at 37 °C to solidify the hydrogels. Cells were seeded at a density of 7500 cells/well on the solidified Matrigel beds and grown for 11 days in standard culture conditions. Cultures received fresh medium supplemented with 2% Matrigel on days 8, 11, and 14. The 3D adherent cultures are referred to as microtumors, which is in agreement with a previous publication [24]. The growth and size of the microtumor cultures was previously investigated [24,25], reporting MIA PaCa-2 microtumor sizes of 3.3 × 10^4^ ± 1.2 × 10^3^ µm^2^ on day 11 and 3.6 × 10^4^ ± 1.2 × 10^3^ µm^2^ on day 15.

### 2.8. Assessment of Treatment Effects in 3D Cultures

PDT and chemotherapy were initiated on day 11. For PDT, cultures were incubated for 1 h with 0.25 µM BPD, after which the medium was refreshed and cultures were exposed to 690-nm laser light (Intense Ltd., North Brunswick, NJ, USA) at an irradiance of 150 mW/cm^2^ and a radiant exposure of 25 J/cm^2^. For chemotherapy, cultures were exposed to 500 µM oxaliplatin during a period of 72 h (day 10–13); after the 72-h incubation, the medium was refreshed. Redox imaging was performed, as described above, on culture day 11 (pre-treatment) and culture day 15 (post-treatment) on the same cultures. 

Following redox imaging, microtumor size (area), viability, viable area, and fractional viable area were determined for all individual microtumors using live/dead staining [26], confocal laser scanning microscopy, and a subsequent analysis performed with the CALYPSO image analysis methodology that was elaborately described before [21,24,25,27].

### 2.9. Analysis of Redox Metabolism on Ex Vivo Tissue Slices

All animal experiments were performed in compliance with the Institutional Animal Care and Use Committee of Massachusetts General Hospital (MGH, protocol number 2005N000284), and as described previously [28]. Male Swiss nu/nu mice (COX7 MGH, four weeks old, 20–24 g) received 1 × 10^6^ MIA PaCa-2 cells in 50% growth factor-reduced Matrigel (200 µL injection volume) via injection in the pancreas under ketamine/xylazine anesthesia. Nine days post-implantation, mice were randomly distributed in control and treatment groups (*N* = 3/group). PDT treatment consisted of intravenous administration of 0.25 mg/kg Visudyne (QLT Phototherapeutics, Vancouver, BC, Canada) in 50 µL of phosphate-buffered saline (PBS), a 1-h drug/light interval, and 690-nm irradiation (HPD/Intense, North Brunswick, NJ, USA) at 100 mW/cm^2^ with a radiant exposure of 50 J/cm^2^ under ketamine/xylazine anesthesia. On day 13 (four days post-treatment), animals were sacrificed, and tumor tissues were excised. This time point of four days post-treatment was chosen based on the following rationale: (1) the initial reactive oxygen species (ROS) production by PDT was likely dissipated, allowing potential metabolic effects or prolonged redox imbalances to become more apparent; (2) the effects of the treatment may not have fully materialized, i.e., sufficient tumor material could still be histologically investigated.

Following animal euthanasia and tumor tissue excision, the tissues were embedded in Tissue-TEK Optimal Cutting Temperature compound (VWR, Franklin, MA, USA), and immediately frozen at −80 °C. The cryogenically stored tissues were sliced in a cryomicrotome at a thickness of 10 µm and were placed on microscope slides. Following a gentle wash in distilled water and subsequent drying for 20 min at room temperature (in accordance with procedures previously described [29]), slides were imaged as described in Section 2.5, and NAD(P)H and FAD fluorescence intensities were quantified, with the only adaptation comprising the acquisition of a 15-image z-stack at a step-size of 0.1 µm. Image analysis was performed as described in Section 2.6. Heatmaps were manually stitched to reconstruct the tissue slice after processing, and the median redox ratio per image was taken as the primary output. Hematoxylin and eosin (H&E) staining was performed using standard protocols.

### 2.10. Statistical Analysis

Statistical analysis was performed using Graphpad Prism 5.0 (La Jolla, CA, USA). Data distributions were tested for normality using a D’Agostino and Pearson omnibus test. As indicated in the figure legends, normally distributed datasets were analyzed using a one-way ANOVA and Bonferroni post hoc test for multiple comparisons, whereas non-Gaussian data sets were analyzed using a Kruskal-Wallis and Dunn’s post hoc test for multiple comparisons. Statistical significance is indicated by a single, double, triple, or quadruple asterisks (for *p* ≤ 0.05, *p* ≤ 0.01, *p* ≤ 0.005, and *p* ≤ 0.001, respectively).

## 3. Results

### 3.1. Analysis of Redox Metabolism in 3D Culture Models

Although redox imaging of organotypic cultures was demonstrated using custom-built optical setups to derive a comprehensive and informative optical metabolic index [10,11], such instrumentation is not typically available in biological laboratories. Intensity-based redox imaging is compatible with conventional microscopes at high magnifications [12,14], and it was elegantly demonstrated to correlate well with biochemically determined redox ratios in two-dimensional (2D) monolayer cultures [14]. For its application to 3D cultures, we adapted established intensity-based redox imaging to function at low magnifications. As two-photon microscopy is prone to cause photothermal tissue damage and photobleaching [30,31], we summed 30 repetitive scans that were taken over a small 0.1-µm z-stack, resulting in a 2D image. An automated image analysis tool was developed to identify spheroid/microtumor outlines, perform background extractions, generate redox ratio heatmaps, and extract ORRs for each spheroid/microtumor from the images (Figure 1). The imaging and image analysis protocols were carefully optimized to correct for spectral overlap, ensure data acquisition within the dynamic range of the detectors, and verify the subcellular origin of the fluorescent signals (Figure S1).

**Figure 2:**
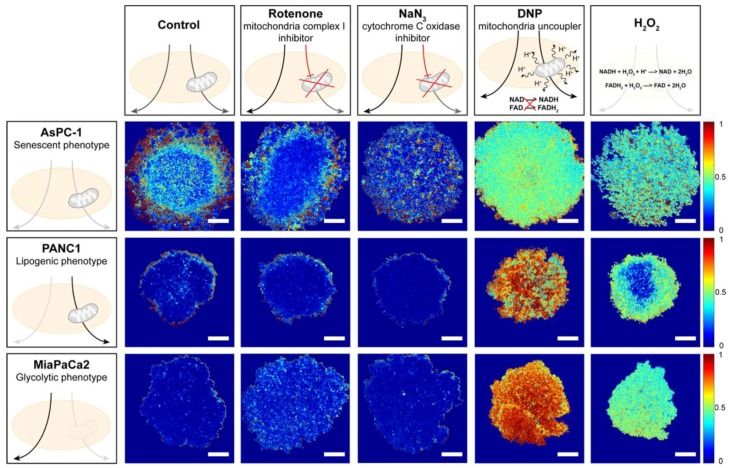
Proof of concept of the redox imaging protocol; image analysis demonstrates the metabolic differences between spheroids of varying pancreatic cancer cell lines. Image analysis outputs demonstrating the optical redox ratio (ORR) spatial distribution throughout the tumor spheroids of PanCa cancer cell lines following exposure to modulators of the reduced nicotinamide dinucleotide (NAD(P)H) and oxidized flavoprotein adenine dinucleotide (NAD(P)H/FAD) balance as illustrated. Scale bar = 250 µm.

**Figure 3:**
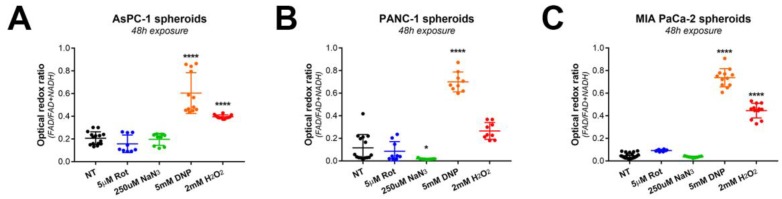
Proof of concept for the redox imaging workflow to detect ORR changes in pancreatic ductal adenocarcinoma (PDAC) spheroids under controlled conditions. Quantification of ORR in spheroids of AsPC-1 (**A**), PANC-1 (**B**), and MIA PaCa-2 (**C**) cell lines. Spheroids were either untreated (black), or imaged following 48-h exposure to 5 µM rotenone (blue), 250 µM sodium azide (NaN_3_) (green), 5 mM 2,4-dinitrophenol (DNP; orange), or 2 mM H_2_O_2_ (red). Black horizontal lines indicate the median optical redox ratio per group. Data were obtained from *N* = 6–9 samples from three experimental repeats. * *p* ≤ 0.05, **** *p* ≤ 0.001.

To determine whether redox imaging can distinguish between cancer spheroids with different metabolic phenotypes, a control experiment was performed on spheroid cultures of metabolically characterized PDAC cell lines, namely, AsPC-1 (senescent phenotype), PANC-1 (lipogenic phenotype), and MIA PaCa-2 cells (glycolytic phenotype) [32]. ORR-based metabolic imaging supported the different metabolic phenotypes of these cell lines, with AsPC-1 spheroids exerting the highest redox states (median 0.21 ± 0.06, Figure 2 and Figure 3A), which were significantly elevated compared to PANC-1 and MIA PaCa-2 spheroids (median ORR 0.12 ± 0.12 and 0.05 ± 0.02, respectively). For the MIA PaCa-2 and PANC-1 spheroids, low redox states were consistent with their lipogenic and glycolytic phenotypes, respectively (Figure 2 and Figure 3B,C). When exposed to the mitochondrial complex I inhibitor rotenone, AsPC-1 spheroids, but not PANC-1 and MIA PaCa-2 spheroids, demonstrated substantially reduced redox states (median ORR 0.16 ± 0.07, *p* = 0.08) (Figure 2 and Figure 3). However, exposure to the cytochrome C inhibitor NaN_3_ [33] did not reduce the ORR (Figure 2 and Figure 3). In addition, exposure to compounds that are known to elevate redox states increased the ORR in all spheroid types, with 2,4-dinitrophenol (DNP, a mitochondrial uncoupler, median ORR > 0.5) being slightly more effective than the oxidizing agent H_2_O_2_ (median ORR range 0.2–0.5; Figure 2 and Figure 3). All imaging procedures and treatments were well tolerated in the spheroid cultures with the exception of H_2_O_2_ (Figure S2).

### 3.2. Cancer Therapies Alter Redox States in Adherent Microtumor Cultures of PDAC

As an alternative to suspended spheroids, cancer cells can be grown as adherent microtumor cultures using extracellular matrix scaffolds [24]. Such models are frequently utilized in investigations on cancer therapy responses [25,34,35], patient-derived cancer organoids [36,37], and oncogenic transformation mechanisms [38,39,40]. For redox imaging, such models are interesting as tens to hundreds of microtumors can be imaged simultaneously within a single experiment, which can be rapidly analyzed with automated image analysis to yield multiparametric readouts. By leveraging this capacity for high-content screening of redox metabolism, we explored the effects of photodynamic therapy (PDT) and oxaliplatin chemotherapy on adherent PDAC spheroids. Whereas PDT is a light-activated cancer therapy that locally generates high levels of reactive oxygen species (ROS) and causes cytotoxic oxidative damage to cellular constituents [41,42,43,44], oxaliplatin chemotherapy inhibits cancer growth by crosslinking DNA, inducing double-strand breaks and apoptotic cell death [45]. These two mechanistically distinct treatments achieve promising results in the clinical management of PDAC [46,47].

**Figure 4:**
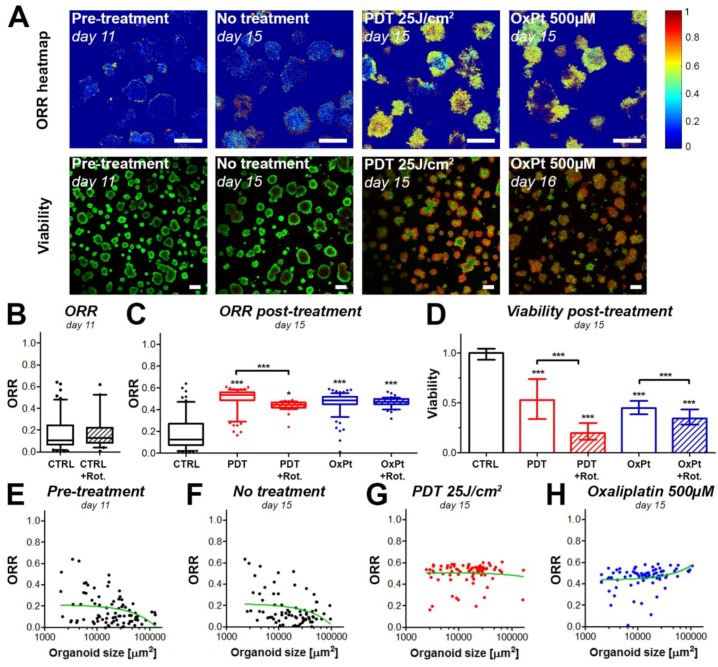
The metabolic redox state in MIA PaCa-2 microtumors changes in response to photodynamic therapy (PDT) and chemotherapy. (**A**) ORR heatmaps and live/dead fluorescence images (live cells in green, dead cells in red) of the same cultures are depicted before and after treatment. Scalebar = 100 µm. (**B**) The box-whisker plot displays the median, 25th and 75th percentile, and the 90% confidence interval of the microtumor populations. (**C**) Quantification of the ORR of MIA PaCa-2 microtumors post-treatment (PDT or oxaliplatin chemotherapy (OxPt) with or without rotenone (Rot)). The box-whisker plot depicts the median, 25th and 75th percentile, and the 90% confidence interval of the microtumor populations. (**D**) As an auxiliary assay to the ORR determinations, viability of the microtumors was assessed post-treatment. The bars represent the mean ± 95% confidence interval of the microtumor populations. (**E–H**) ORR-size correlations are shown for the microtumors pre-treatment on day 11 (**E**), or on day 15 for the untreated control group (**F**), the 25 J/cm^2^ PDT-treated group (**G**), and the 500 µM oxaliplatin-treated group (**H**). In the scatter plots, the linear regression curve fit (in green) provides a qualitative indication of the size-ORR correlation. Data presented in panels **A** and **E–H** are from a single representative experiment, whereas the data plotted in panels **B–D** were obtained from 2–3 technical repeats with *N* = 3 wells containing ~700 individual microtumors/well. * *p* ≤ 0.05, *** , *p* ≤ 0.005.

For these investigations, the MIA PaCa-2 cell line was selected as we recently published the dose-response effects of both PDT and oxaliplatin [25]. Prior to treatment, MIA PaCa-2 microtumors displayed low redox ratios both in the presence and absence of 5 µM rotenone (Figure 4A,B), which corroborated the observations on the suspended microtumors. A slight negative correlation was observed between the ORR and the microtumor size (Figure 4E), i.e., the smaller the microtumors, the higher the ORR. Examining the corresponding heatmaps (Figure 4A) reveals that the redox states were more elevated around the periphery of the cancer microtumors, thus explaining that larger microtumors (with higher core/periphery ratios) have a slightly elevated median ORR (Figure 4E). 

Treatment of adherent MIA PaCa-2 microtumors with PDT resulted in a significant increase in ORR (Figure 4C) and a decrease in viability (Figure 4D). These findings corroborrate previous studies in which PDT raised redox states in cancer tissues by inducing severe oxidative stress [18,19]. Heatmaps of ORR and viability showed similar patterns, where areas of low viability and high ORR were observed in small microtumors and at the periphery of larger microtumors. The median redox states were relatively homegeneously dispersed amongst the microtumors regardless of their size, with a slight trend for larger microtumors to have a lower redox state (Figure 4F). In comparison to PDT alone, the ORR was slightly yet significantly reduced following treatment with both PDT and rotenone. This observation suggests that OxPhos may contribute in part to the increased ORR in PDT-treated microtumors, but that the main cause for the highly oxidative states is likely of non-metabolic origin. In addition, there was a significant reduction in viability when MIA PaCa-2 microtumors were treated with PDT + 5 µM rotenone compared to PDT alone. This, in combination with the reduced redox ratio, suggests that cells that survive the initial oxidative insult by PDT may potentially resort to OxPhos to facilitate their survival, a process that is prohibited by blocking mitochondrial complex I with rotenone.

Following oxaliplatin chemotherapy, the median microtumor ORR was similar to the PDT-treated group, albeit with lower intra-microtumor heterogeneity (Figure 4C). Similar to the PDT treatment, viability was substantially decreased (median viability = 0.45, Figure 4D) yet homogeneously dispersed throughout the cancer microtumors. When additionally incubated with 5 µM rotenone, the median ORR was not affected, suggesting that the increased redox ratio primarily originates from reduced microtumor health (e.g., mitochondrial uncoupling). The slight yet significant reduction in viability following exposure to 5 µM rotenone + 500 µM oxaliplatin compared to 500 µM oxaliplatin alone was attributed to the mild toxicity of rotenone which may cause a slight additive effect (Figure S2), altogether suggesting that OxPhos has no major function in oxaliplatin resistance. With respect to the ORR-size correlation, there was a minor positive correlation, demonstrating that larger microtumors exhibited a higher ORR following oxaliplatin therapy (Figure 4F–H).

### 3.3. Redox States of Pancreatic Tumors are Affected by Photodynamic Therapy In Vivo

**Figure 5:**
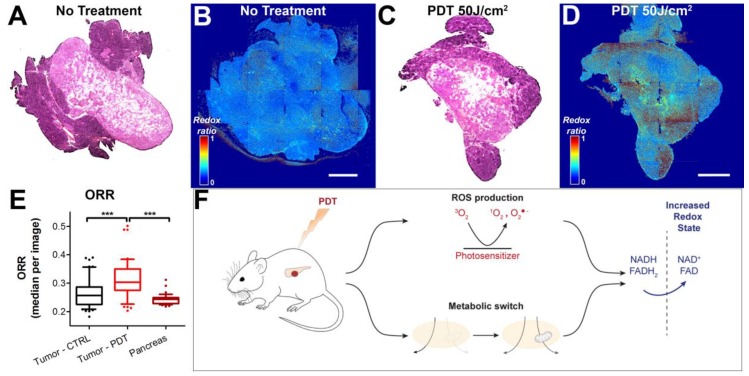
Redox imaging on ex vivo tissue slices of orthotopic MIA PaCa-2 tumors extracted four days post-PDT demonstrates an ORR increase following PDT. Scalebar = 1 mm. Tissue slices of orthotopically implanted MIA PaCa-2 tumors were stained with hematoxylin and eosin (H&E). Representative samples of the no treatment control group (**A**) and the PDT-treated group (**C**) are represented. Stitched redox ratio heatmaps of adjacent tumor slices are represented in (**B**) and (**D**) for the untreated tumor and the PDT-treated tumor, respectively. (**E**) Quantification of the ORR is shown in the box-whisker plots, in which the median, 25th and 75th percentiles, and 90% confidence interval of the median redox ratio per image are plotted. Data pertaining to the untreated tumor tissue are depicted in black (*N* = 3 tumors, 51 images), whereas data pertaining to the PDT-treated tumor tissue are represented in red (*N* = 3 tumors, 49 images). (**F**) Schematic representation of the potential origin of the redox perturbations. *** *p* ≤ 0.005.

To determine whether the effects observed in the in vitro 3D cultures are recapitulated in vivo, we subjected mice carrying orthotopic MIA PaCa-2 xenografts to a sublethal dose of PDT (50 J/cm^2^) using 0.25 mg/kg Visudyne as a photosensitizer. Tumors were harvested four days after treatment, and subsequently imaged using established methods for ex vivo redox imaging [29]. The H&E stains emphasize clearly demarcated tumor tissue, with some surrounding pancreatic tissue that was more intensely stained with hematoxylin (Figure 5A,C). On the redox images, however, the tumor tissue could not easily be distinguished from the pancreatic tissue. On the ORR heatmaps, the controls (no treatment) demonstrated a homogeneously dispersed redox ratio (median 0.26 ± 0.05, Figure 5B,E). Following treatment, the ORR heatmaps showed heterogeneous regions with high redox states. The average ORR in these tumors was significantly higher compared to the no treatment controls (*p* < 0.005), and had an average of 0.31 ± 0.07 (Figure 5D,E). Taking into account the findings on adherent microtumor PDAC cultures, the results suggest that elevated redox states are caused by treatment-induced oxidative stress and a metabolic transition in the cancer tissues (Figure 5F). Together, these observations demonstrate that changes in redox state on ex vivo tissue slices corroborate the results obtained in vitro. It provides a proof of concept that the redox imaging and automated image analysis workflow can be employed in translational studies to investigate the effects of treatment on cancer metabolism.

## 4. Discussion

The aberrant metabolism of PDAC is increasingly associated with the treatment-resistant nature of this disease. In particular, recent discoveries revealed that PDAC relies on aberrant autophagy, glycolysis, and glutaminolysis [2], although the exact pathways that are implicated in therapy resistance remain largely elusive. Given the resistant nature of PDAC, there is an ongoing effort to identify therapeutic combinations that can augment the clinical standard of care, for which oxaliplatin is frequently involved as part of the FOLFOX (folinic acid, 5-fluorouracil, oxaliplatin) or FOLFIRINOX (folinic acid, 5-fluorouracil, irinotecan, oxaliplatin) regimens. In addition, given that BPD-PDT is under clinical evaluation for PDAC, understanding its effect on cancer metabolism is becoming increasingly important. Therefore, in this study, we explored the use of redox imaging to potentially identify alterations in oxidative metabolism as a result of these clinically relevant treatments. We outlined the adaptation of intensity-based redox imaging with conventional fluorescence microscopy and presented a workflow for unbiased high-throughput image analysis. We corroborated earlier studies in which increased redox states were observed following PDT (further discussed below). The method also revealed that oxaliplatin induces a similar increase in redox state on MIA PaCa-2 cancer microtumors. However, in contrast to oxaliplatin, inhibition of oxidative phosphorylation using rotenone caused a slight yet significant decrease in redox state and a strong reduction in viability following PDT. These findings suggest that the glycolytic MIA PaCa-2 cells may rely on oxidative metabolism in order to survive BPD-PDT. Secondly, the results imply that the major cause for the BPD-PDT- and oxaliplatin-induced oxidative states is of a non-metabolic origin and, thus, likely stems from mitochondrial uncoupling prior to apoptosis.

We recently demonstrated that the redox imaging workflow presented here can be used to uncover the effect of stromal cells on cancer metabolism using 3D heterotypic microtumor models of PDAC. These models were developed as stromal partner cells as these can influence cancer metabolism and treatment responses [48,49,50]. The heterotypic microtumors were composed of PDAC cells that were grown in direct co-culture with cancer-associated fibroblasts (CAF6 cells) [51,52]. The redox imaging workflow revealed that, in absence of treatment, the CAFs elevated the basal redox states in the PDAC microtumors and induced pro-survival proteins such as heme oxygenase 1 and cyclooxygenase 2 [24]. However, redox changes post-treatment were not further investigated in that study. The unique focus of the current paper is to present the practical details and validation this redox imaging workflow, provide further indications for its application to preclinical cancer models, and present it as a feasible and low-cost solution for investigations toward the identification of treatment-induced perturbations in redox state and cancer metabolism.

Although redox imaging was explored for quite some time, it was applied mostly to conventional 2D cultures or histological preparations. Intensity-based redox imaging works well in these contexts as high magnifications can be used to capture the relatively low fluorescence intensities emitted by the endogenous fluorophores. For example, Pogue et al. demonstrated that BPD-PDT led to a light dose-dependent decrease in NAD(P)H autofluorescence in radiation-induced fibrosarcoma-1 (RIF-1) cells [19]. Subsequent in vivo measurements confirmed that BPD-PDT of muscle tissue reduced NAD(P)H autofluorescence, whereas the fluorescence lifetime of NAD(P)H (i.e., protein-bound state) remained largely unaffected, suggesting that PDT reduces tissue NAD(P)H concentrations [19]. Similar findings were obtained by Zhang et al. [18], who demonstrated that PDT with the photosensitizer pyropheophorbide-2-deoxyglucosamide induced significantly increased redox states in cryosections of rat 9L glioma tissue. In a seminal study by Skala et al., in vivo multiphoton microscopy was used for integrated fluorescence intensity and lifetime imaging of NAD(P)H and FAD in precancerous epithelium [15]; 3D-resolved redox imaging revealed decreased redox ratios in precancerous lesions compared to healthy epithelium. In addition, the investigators noted significant reductions in protein-bound NAD(P)H and FAD using fluorescence lifetime imaging that was dependent on the grade of dysplasia in the epithelial lesions [15]. These studies clearly demonstrated that an abundance of metabolic information on cancer metabolism and treatment-induced aberrations can be obtained using endogenous contrast imaging. 

Recent years also saw the emerging application of redox imaging to various forms of 3D cultures, of which three examples are given below. Firstly, Walsh et al. used a custom-built multiphoton microscopy setup for fluorescence lifetime imaging of NAD(P)H and FAD on patient-derived breast cancer and pancreatic organoids [10,53]. By integrating the redox ratio with the fluorescence lifetimes of NAD(P)H and FAD, this approach was capable of identifying redox changes in response to paclitaxel, gemcitabine, and various molecular inhibitors including pilaralisib [10,53]. A study by Chang et al. used a custom-built microscopy set-up for combined two-photon-excited fluorescence imaging and third-harmonic generation imaging to investigate the relationship between redox state and lipid droplet formation in mesenchymal stem cells [54]. This was investigated on a 3D model that mimicked adipose tissue, which consisted of mesenchymal stem cells embedded in silk scaffolds. With a comprehensive image analysis workflow, they identified reduced redox state and increased volumes of lipid droplets in cells exposed to adipogenic media, indicative of cell differentiation [54]. More recently, Madonna et al. presented an alternative metabolic imaging strategy on murine mammospheres [55]. A conventional confocal microscopy setup was used to image 2-[*N*-(7-nitrobenz-2-oxa-1, 3-diaxol-4-yl) amino]-2-deoxyglucose (2-NBDG) and tetramethyl rhodamine ethyl ester (TMRE) as molecular probes for glucose uptake and mitochondrial membrane potential, respectively. This method revealed metabolic alterations in mammospheres representing different disease stages of breast cancer, showing a global decreases in glucose uptake and increases in mitochondrial membrane potential as the disease stage progressed [55]. These studies demonstrated the power of metabolic imaging on various 3D culture models, but also highlighted that NAD(P)H/FAD-based redox imaging on such cultures with conventional microscopy setups is highly challenging.

Compared to these previous studies exploring redox imaging on 3D culture models, a major benefit of the approach presented in this study is that it provides a wealth of information using widely available instrumentation and established image analysis tools. Although none of these aspects are new concepts in their own respect, their integration into an easily implementable bioassay was not previously explored. Leveraging this method to obtain multiparametric readouts for numerous individual microtumors per sample enabled us to compare basal redox states and treatment-induced perturbations in a statistically robust manner. However, redox imaging by itself, regardless of the method used, is rather inconclusive, as redox perturbations can have multiple origins. Continued research should, therefore, leverage redox imaging as a multiparametric non-perturbative method that can be easily combined with, and corroborate, more specific metabolic assays (e.g., glucose/lactate quantification [56], metabolic flux analysis [57], ROS detection [58]). 

A limitation of the current method is that we cannot identify redox perturbations on a single-cell level. Naturally, this is the consequence of using the low magnifications, which augments the throughput of the method with respect to whole microtumor/spheroid imaging, at the expense of resolution. The addition of fluorophores such as cell tracker dyes is not recommended given the relatively low fluorescence emission of NAD(P)H and FAD: The emission of auxiliary fluorophores will likely dominate over the low fluorescence emission of these cofactors. One possible solution, which remains to be explored, is the sectioning of individual organoids, on which the redox imaging can be done as described for the in vivo experiment. In sequential sections, one can investigate the localization of stromal cells and cancer cells using immunohistochemistry, and investigate the redox state of the tissue using the redox imaging method on subsequent slides. However, such approaches are end-point measurements, and dynamic changes in redox state will be difficult to capture.

One potential application of redox imaging that remains to be further explored is the preparation of 3D reconstructions of tumor redox states to identify basal and treatment-influenced redox states. Although higher-throughput imaging methods are desirable for such applications, there are in principle no technical constraints preventing to obtain full 3D reconstructions of the redox state in spheroids, organoids, microtumors, and tumor tissues in vivo and ex vivo. For real-time imaging in vivo, the obvious limitation remains the limited optical permeability of tissue. For example, in vivo multiphoton microscopy was demonstrated to enable fluorescence lifetime imaging of NAD(P)H and FAD up to a depth of 70 µm [15]. With two-photon microscopy, the image acquisition may be possible at a depth of up to 1 mm [59,60]. As such, it should be kept in mind that full 3D reconstructions of large tumor masses can only be acquired ex vivo, thus limiting the ability to capture dynamic changes in redox state. For ex vivo redox imaging, the use of fiducial markers is recommended, especially when interested in correlating redox changes to treatment response. This approach could identify correlations between redox changes and experimental parameters such as the extent of drug diffusion (e.g., chemotherapeutics, photosensitizers) or the direction and diffusion of excitation light throughout the tumor tissues.

With the advent of advanced microscopy tools such as adaptive optics and light-sheet microscopy, perhaps both single-cell and full tumor evaluations within 3D and in vivo cancer models and patient tissues will become more widely available in the near future. Combinations of the different methods presented by us and others may ultimately pave the way for these investigations. Such methods, potentially in combination with other spatially and/or temporally resolved metabolic imaging methods will be instrumental to optimize therapeutic and diagnostic strategies and advance cancer management [61].

## 5. Conclusions

Tracking the adaptive metabolism and redox state in organotypic cultures and ex vivo cancer tissues represents a powerful investigational tool that can be easily combined with complementary assays to assess treatment effects in a more comprehensive manner. The results of this study revealed metabolic alterations in both adherent microtumor cultures and suspended spheroid cultures derived from three PDAC cell lines. The PDT-induced perturbations in redox state were recapitulated on an orthotopic pancreatic cancer model in vivo. Spatiotemporal tracking of metabolic states in cancer tissues will provide a deeper understanding on metabolic plasticity in malignant tissues and aid in elucidating the metabolic origins that lead to therapy insensitivity. Such discoveries will ultimately facilitate the design of new mechanistically informed cancer therapies. Such advances are particularly relevant in the clinical management of advanced pancreatic cancer, as there remains an urgent need for more effective and less harmful treatment strategies. 

## Supplementary Materials

The following are available online at https://www.mdpi.com/2077-0383/8/9/1399/s1: Figure S1: Determination of the spectral overlap, origin, and dynamic range of NAD(P)H and FAD fluorescence emission during the redox imaging procedure; Figure S2: Toxicity evaluation for controlled redox states and redox imaging.
