# Supplementary material for: Tracking Photodynamic- and Chemotherapy-Induced Redox-State Perturbations in 3D Culture Models of Pancreatic Cancer: A Tool for Identifying Therapy-Induced Metabolic Changes
Source: J Clin Med. 2019 Sep 6;8(9):1399. doi: 10.3390/jcm8091399 (PMC6788194; doi:10.3390/jcm8091399)
Supplement: Supplementary file 1 [file jcm-08-01399-s001.pdf]

Tracking Photodynamic- and Chemotherapy-Induced Redox-State Perturbations in 3D Culture Models of Pancreatic Cancer: A Tool for Identifying Therapy-Induced Metabolic Changes

Supplemental Figures

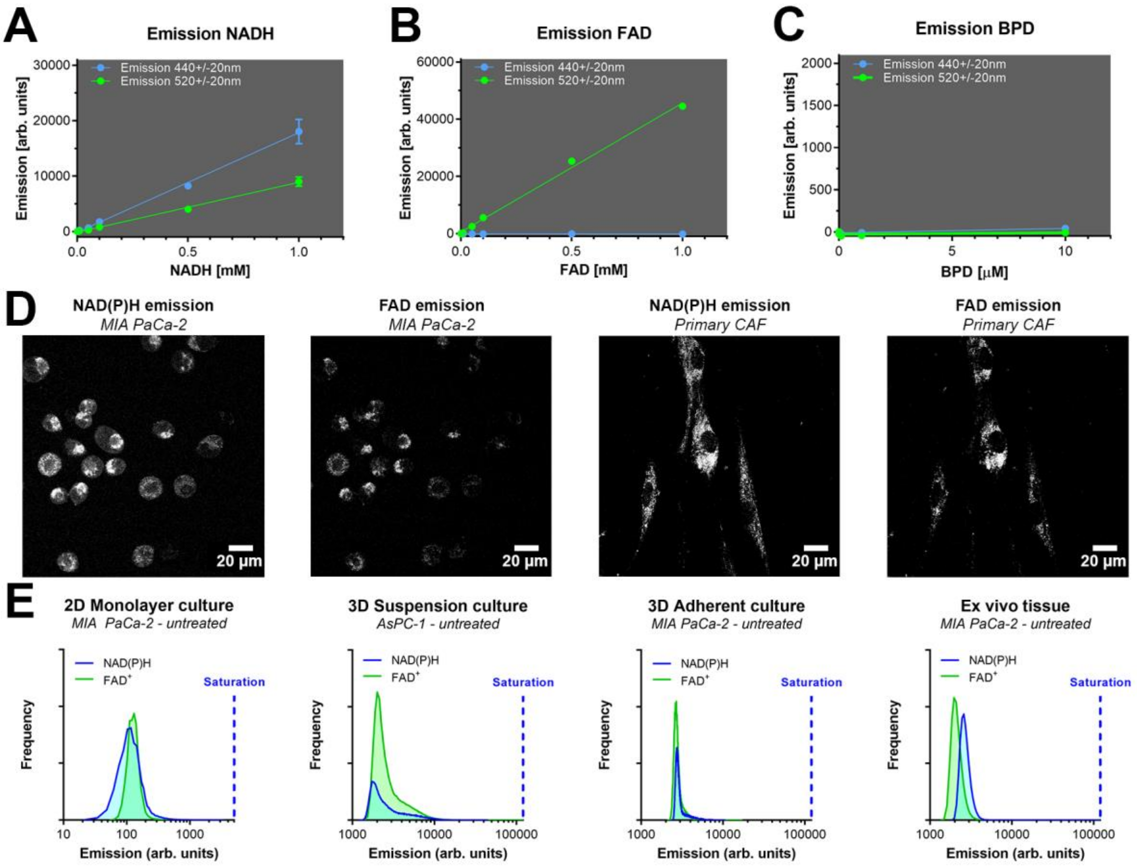

Figure S1. Determination of the spectral overlap, origin, and dynamic range of NAD(P)H and FAD fluorescence emission during the redox imaging procedure. (**A-C**) Determining the spectral overlap of the endogenous fluorophores and cancer therapeutics to ensure correct image analysis. Emission intensity of (**A**) pure NADH, (**B**) pure FAD, and (**C**) BPD solutions at 440 ± 20nm (blue) and 520 ± 20nm (green) upon 750nm excitation. (**D**) Fluorescence emission of NAD(P)H and FAD is mainly detected in perinuclear foci in both MIA PaCa-2 PDAC cells (left panels) and cancer associated fibroblasts (CAF, right panels). Scalebar = 20µm. (**E**) Histograms depicting the tissue-derived emission intensities within the dynamic range of the detector. Histograms depict fluorescence intensities measured in 2D culture, 3D suspended spheroids, 3D adherent spheroids, and ex-vivo cryopreserved tissues.

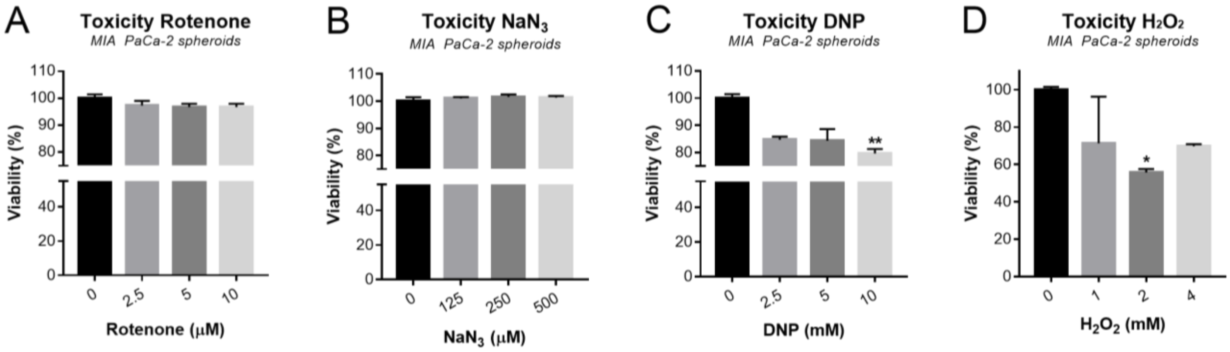

Figure S2. Toxicity evaluation for controlled redox states and redox imaging. (**A**–**D**) Concentration-dependent toxicity of treatments for controlled redox states were determined on MIA PaCa-2 spheroids for (**A**) rotenone, (**B**) NaN_3, (**C**) dinitrophenol (DNP), and (**D**) H_2O_2. Statistically significant differences are indicated between treatment groups versus the control group. Statistical analysis was performed using a Kruskal-Wallis/Dunn's multiple comparisons test. * $p \leq 0.05$, ** $p \leq 0.01$.
